# Transcriptome sequencing of a thalloid bryophyte; *Dumortiera hirsuta* (Sw) Nees: assembly, annotation, and marker discovery

**DOI:** 10.1038/srep15350

**Published:** 2015-10-20

**Authors:** Harpal Singh, Krishan Mohan Rai, Santosh Kumar Upadhyay, Poonam Pant, Praveen Chandra Verma, Ajit Pratap Singh, Pradhyumna Kumar Singh

**Affiliations:** 1 CSIR-National Botanical Research Institute, Council of Scientific and Industrial Research, Rana Pratap Marg, Lucknow 226001, UP, India; 2 Department of Botany, Kashyap Block, Panjab University, Chandigarh, 160014-UT, India

## Abstract

Bryophytes are the first land plants but are scarcely studied at the molecular level. Here, we report transcriptome sequencing and functional annotation of *Dumortiera hirsuta*, as a representative bryophyte. Approximately 0.5 million reads with ~195 Mb data were generated by sequencing of mRNA using 454 pyrosequencer. *De novo* assembly of reads yielded 85,240 unigenes (12,439 contigs and 72,801 singletons). BlastX search at NCBI-NR database showed similarity of 33,662 unigenes with 10-^10^ e-value. A total of 23,685 unigenes were annotated at TAIR10 protein database. The annotated unigenes were further classified using the Gene Ontology. Analysis at Kyoto Encyclopedia of Genes and Genomes pathway database identified 95 pathways with significant scores, among which metabolic and biosynthesis of secondary metabolite were the major ones. Phenylpropanoid pathway was elucidated and selected genes were characterized by real time qPCR. We identified 447 transcription factors belonging to 41 families and 1594 eSSRs in 1479 unigenes. *D. hirsuta* unigenes showed homology across the taxa from algae to angiosperm indicating their role as the connecting link between aquatic and terrestrial plants. This could be a valuable genomic resource for molecular and evolutionary studies. Further, it sheds light for the isolation and characterization of new genes with unique functions.

Bryophytes are the oldest known terrestrial plants with high ecological and economic value. These are among the highly stress tolerant fauna of the world. Some of them resurrect even after complete dryness. Although several economically important secondary metabolites, anti-fungal, anti-microbial compounds and enzymes are reported from bryophytes, characterization of the later at genetic and molecular levels has not yet been done. Genome and transcriptome data of this group can be helpful in evolutionary and taxonomic studies and can also contribute significantly in the determination of its ecological attributes and economic importance. Even after 8 years of Next Generation Sequencing (NGS) technology being available, genome sequence of only one bryophyte (*Physcomitrella patens*) has been reported till date^1^.

*Dumortiera hirsuta* is a thalloid liverwort reported from all bryo-geographical regions in India. It grows on moist rocks in shady places around the waterfalls^2^ and has been widely used for the isolation of several economically important compounds. The aqueous extract of *D. hirsuta* is effective against several phyto-pathogens^3^. The plant has inherent abilities of stress tolerance^4^. It produces superoxide radicals (a common response to biotic and abiotic stresses) as an extracellular oxidative burst during rehydration after desiccation stress^5^. This leads to the signal transduction and formation of suberin, melanin and lignin like protective substances for defense against phyto-pathogens^6^,^7^,^8^. Different isoforms of peroxidases (EC 1.11.1.7) and tyrosinases (EC 1.10.3.1) are found in the apoplast of *D. hirsuta* and are responsible for the oxidative burst^9^. Despite the economic significance and cosmopolitan occurrence, *D. hirsuta* has not been studied at molecular level. Moreover, it is difficult to study an important metabolic pathway of uncommon/non-model organisms where the reference genome/transcriptome is not available.

High-throughput sequencing is an important step towards the development of nucleotide sequence database of non-model organisms like *D. hirsuta*. *De novo* sequencing of *D. hirsuta* on NGS platform like 454 pyrosequencer will be very useful as it generates longer read lengths which makes the assembly easy and accurate. The sequence data generated by transcriptome sequencing facilitates elucidation of metabolic pathways, global gene expression and many others. Present study describes the transcriptome sequencing of *D. hirsuta* and their assembly to get a non-redundant set of unigenes. The present study also describes the annotation of unigenes with different proteins datasets, their gene ontology, elucidation of important metabolic pathways, identification of transcription factor families and EST-SSRs markers. As *D. hirsuta* has some unique properties, the study is expected to help in designing new strategy for the better utilization of plant resource for mankind.

## Results

### Sequencing and assembly

**Figure 1:**
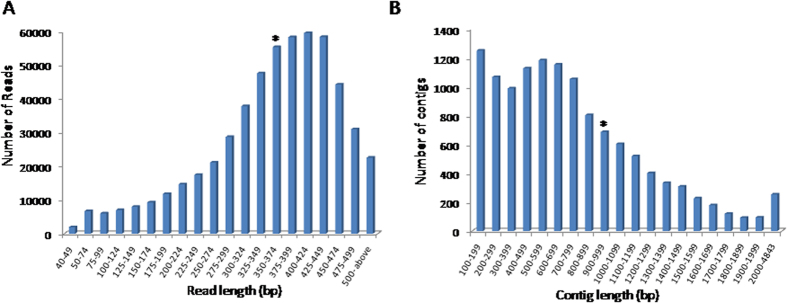
Size distribution of *Dumortiera hirsuta* (**A**) transcriptomic raw reads and (**B**) contigs after assembly. Size groups with average read length and average contig length were shown with asterisk (*) mark over the bar.

*D. hirsuta* cDNA library was prepared from the mRNA of gametophyte and used for sequencing at 454 pyrosequencer. Signal processing of raw images acquisited during sequencing run on gsRunBrowser v2.5.3 generated 5,48,123 reads, having a total of 19,55,92,000 bases (~195.6 Mb). Average read length was 357 bases with length distribution 40 to 737 bases, (Table 1, Fig. 1A). A total of 1,968 reads having read length less than 50 bases and low quality were removed before further analysis. Remaining reads were mapped on pool of plants ribosomal RNAs using gsMapper v2.5.3 and 1, 21,117 reads of ribosomal origin were omitted from the analysis. After the quality filtration, 4, 25,038 reads were passed with 95.44% of Q40plus bases and used for *de novo* assembly. 80.7% reads were aligned to assemble into 12, 439 contigs (having length ≥100 bases); rest19.3% reads were singletons. About 64% contigs (8, 007 out of 12, 439) were classified as large contigs having length ≥500 bases (Table 2, Fig. 1B). The average contig size was 989 bases with largest contig of 4843 bases. However the N50 contig size of assembled data was 1071 bases. All the contigs and singletons (12, 439 and 72, 801, respectively) were pooled together to form 85, 240 non-redundant set of unigenes. The transcriptome sequencing data is submitted to NCBI (SRA2A1172).

### Annotations with different databases

*D. hirsuta* unigenes were searched for their annotation against different databases (Table 3). Total 33, 662 unigenes showed similarity with NCBI non-redundant (NR) protein database in BlastX search with cut-off E-value 10^−10^ (File S1). These unigenes represents 20760 proteins of NR database. Similarly, 23, 685 unigenes were annotated against TAIR10 protein database representing 9, 014 unique TAIR locus IDs (File S2). Unigenes with TAIR10 locus IDs were used for further functional annotation.

### GO annotation and KEGG pathway analyses

**Figure 2:**
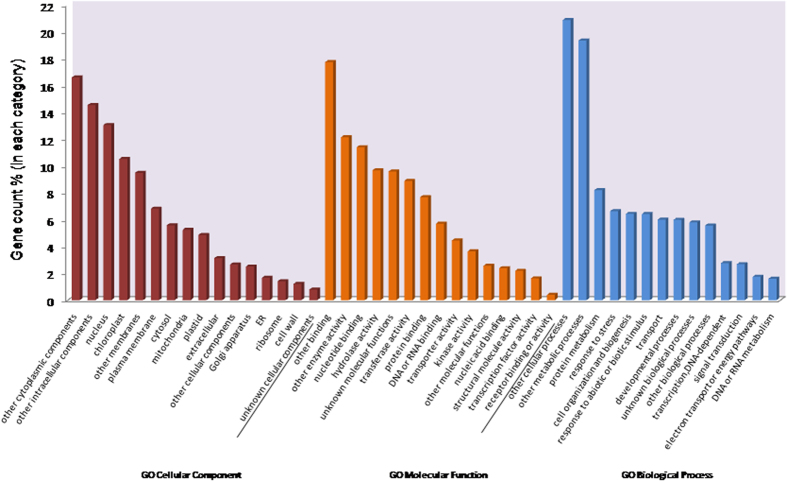
GO annotation details of *Dumortiera hirsuta* unigenes with Tair locus id assigned. All the annotated unigenes are categorized into cellular component (20582 GO terms), molecular function (14493 GO terms) and biological processes (27610 GO terms).

Unigenes with TAIR10 locus IDs were analysed on Gene Ontology to classify them into different GO categories. Out of total 62, 685 GO terms, 20, 582 were assigned to cellular component, 14, 493 to molecular function and 27, 610 to biological processes (Table 3, Fig. 2). Among the cellular components, nucleus (13%) and chloroplast (10.5%) were at the top. Similarly, nucleotide binding (11%), hydrolase (9.7%) and transferease (8.9%) activities were among the most enriched molecular processes. Protein metabolism (8.2%), response to stress (6.6%), cell organisation and biogenesis (6.4%) and response to abiotic and biotic stimulus (6.4%) were among the most enriched biological processes (File S3).

**Figure 3:**
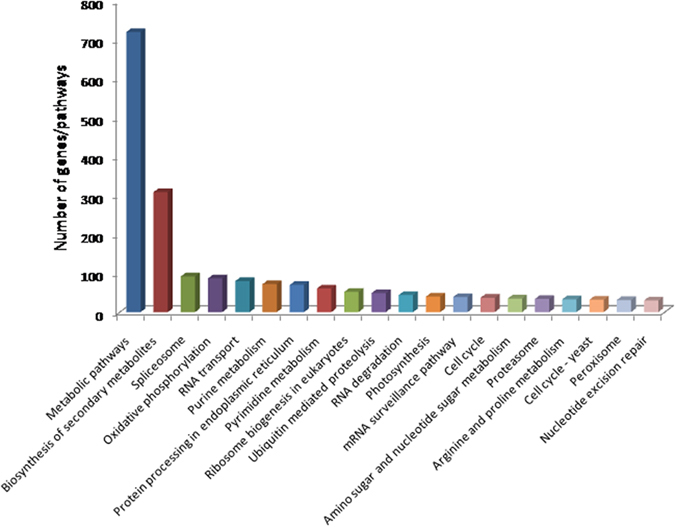
Top 20 identified KEGG pathways. *Dumortiera hirsuta* unigenes with 4305 KEGG Orthologous (KO) ids assigned were search for 95 KEGG pathways.

Unigenes with TAIR locus IDs were searched for KEGG Orthologous IDs and 4305 IDs were identified. These were further searched for various pathways involved in the *D. hirsuta* and 95 significant pathways were identified. Metabolic pathways (comprised of 721 unigenes), biosynthesis of secondary metabolites (309 unigenes), spliceosome (92 unigenes), oxidative phosphorylation (87 unigenes) and RNA transport (80 unigenes) were the top five pathways (Fig. 3, File S4).

### Elucidation of Pathway involved in Stress

Unigenes with TAIR locus IDs were searched for KEGG Orthologous IDs and were mapped for phenylpropanoid pathway. Several important enzymes of this pathway were significantly identified from the unigenes. Cinnamyl-alcohol dehydrogenase (EC: 1.1.1.195) was found to be most expressive with log2 TPM value 7.7, followed by PRDX6; peroxiredoxin 6, 1-Cys peroxiredoxin (EC: 1.11.1.7) with log2 TPM value 7.2. The enzymes with more than 3 log2 TPM value are given in Table 4. Mapped enzymes from the phenyl propanoid pathway (Figure S1) show that most of the important steps have been covered in this study. These results indicate towards the possible important role of phenylpropanoid pathway in the stress tolerance of bryophytes.

**Figure 4:**
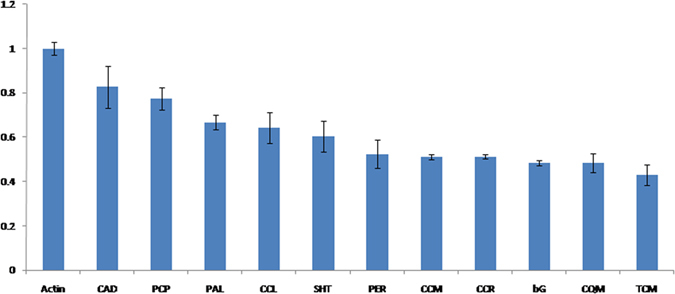
Expression analysis of selected genes of phenyl propanoid pathway. The transcript expression was normalized with the *actin* expression. All the genes analyzed for transcript abundance were found expressing in gametophyte of *D. hirsuta*. The full form of abbreviations given in figure is CAD: Cinnamyl-alcohol dehydrogenase, PCP: Peroxiredoxin 6, 1-Cys peroxiredoxin, PAL: Phenylalanine ammonia-lyase, CCL: 4-coumarate-coA ligase, SHT: Shikimate O-hydroxycinnamoyl transferase, PER: Peroxidase, CCM: Caffeoyl-coA O-methyltransferase, CCR: Cinnamoyl-coA reductase, bG: Beta-glucosidase, CQM: Coumaroylquinate 3′-monooxygenase, TCM: Trans-cinnamate 4-monooxygenase.

Expression analysis of genes involved (log2TPM >3) in phenylpropanoid pathway was analysed by real time PCR. Expression level was compared with the actin gene. We found that all the genes were expressed in gametophyte of *D. hirsuta* (Fig. 4). Significant transcript abundance was observed for each gene when compared to the expression level of actin.

### Transcription Factors

**Figure 5:**
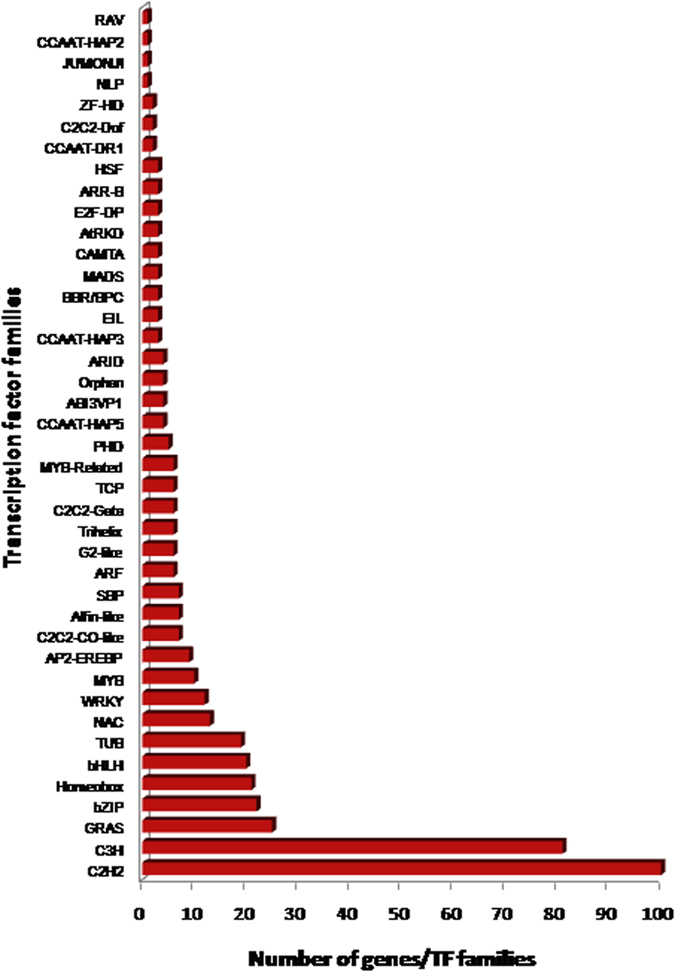
Distribution of identified transcription factors from *Dumortiera hirsuta* unigenes into transcription factor families. C2H2 (100 genes), C3H (81), GRAS (25), bZIP (22) and Homeobox (21) class of TFs were among the top five.

Among the *D. hirsuta* unigenes, a total of 447 transcription factor encoding genes (TFs) were identified (File S5A) with the help of *A. thaliana* transcription factor database (AGRIS). All the TFs were further classified into 41 families (File S5B). C2H2 (100 unigenes), C3H (81 unigenes), GRAS (25 unigenes), bZIP (22 unigenes) and Homeobox (21 unigenes) were among the top five families. bHLH, TUB, NAC, WRKY and MYB TF families were also found present (Fig. 5).

### Similarity with other plant genomes

**Figure 6:**
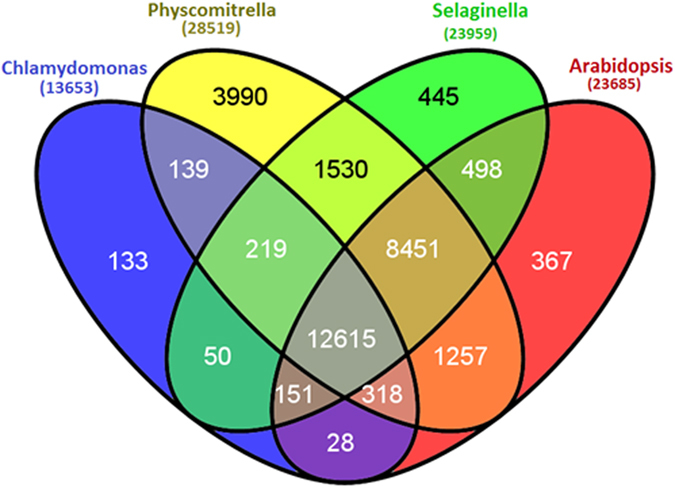
Venn diagram showing the similarity of *Dumortiera hirsuta* unigenes with plant species from four different phyla. *Chlamydomonas reinhardtii* (Blue green algae), *Physcomitrella patens* (Bryophyte), *Selaginella moellendorffii* (Pteridophyte) and *Arabidopsis thaliana* (Angiosperm) total proteins were searched for similarity with *D. hirsuta* unigenes. Number of *D. hirsuta* unigenes having similarity with different plant species proteins were shown in parenthesis.

*D. hirsuta* unigenes were analysed for the pattern of genes distribution across the representatives of four phyla, *C. reinhardtii* (Blue green algae), *P. patens* (Bryophyte), *S. moellendorffii* (Pteridophyte) and *A. thaliana* (Angiosperm) with total number of proteins 19526, 38354, 22285 and 35386 respectively (Fig. 6). Proteins sequences of these plants were downloaded from Phytazome server (http://www.phytozome.net/) and compared with *D. hirsuta* unigenes. A total of 13653 unigenes with *C. reinhardtii*, 28519 with *P. patens*, 23959 with *S.* m*oellendorffii* and 23685 with *A. thaliana* were found similar. A total of 12615 unigenes (representing 14.8%) were found present across all the four genera used to perform this analysis.

A total of 3990 unigenes were found uniquely in *P. patens*. Similarly 133, 445 and 367 unigenes were also found unique to *C. reinhardtii*, *S. moellendorffii* and *A. thaliana*, respectively. Out of total, 1672 unigenes did not have any similarity to *P. patens*, the closest reported genome of a bryophyte.

### Identification of EST-SSRs

**Figure 7:**
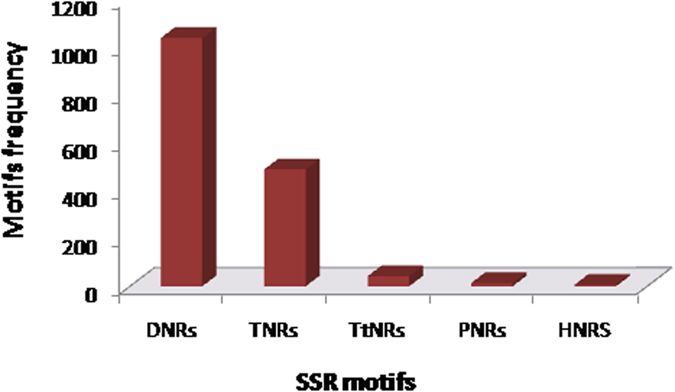
Frequency distribution of 1594 EST-SSRs motifs identified in *Dumortiera hirsuta* unigenes. DNRs (1040; 65%) were among the most abundant EST-SSRs identified (with MNRs excluded).

A total of 81,838 *D. hirsuta* unigenes with more than 100 bases of length were used for EST-SSRs mining using MIcro SAtellite (MISA) identification tool with standard criteria of 5 motifs depth. After excluding mono-nucleotide repeats from the analysis, 1594 SSRs have been identified within 1479 unigenes. Out of total, 102 unigenes were found having more than one SSRs whereas 89 SSRs were found in compound formation *i.e.* within 100 bases of proximity. Among the identified SSR motifs, 1040 DNRs (65.2%), 490 TNRs (30.7), 43 TtNRs, 14 PNRs and 7 were HNRs (Table S1, Fig. 7). AG/CT with 601 motifs in DNRs (57.8% of DNRs) and AGC/CTG with 131 motifs in TNRs (26.7% of TNRs) were the most abundant motifs (File S6).

## Discussion

Here we report the transcriptome analysis of the bryophyte *D. hirsuta.* Bryophytes occupy an important phylogenetic position between aquatic and land plants. They are largely divided into three sub-groups- liverworts, hornworts and mosses. *D. hirsuta* is a rare liverwort, grows in shady and humid rocks. Although genome sequence of moss *P. patens* is reported^1^, the NGS data of a liverwort, the first land plants are expected to be of much more relevance and expected to unveil some of the evolutionary mysteries. Further, they are wonderful examples of stress tolerance. They might have unique pathways for adaptation in aquatic and terrestrial mode of life. Their gene pool might be useful in exploring/modifying the crops for stress tolerance.

A total of 85,240 unigenes identified in the present study shows development of comprehensive NGS data for *D. hirsuta*. We have identified 33,662 unigenes through NCBI-Nr protein database and 23,685 unigenes through TAIR10 protein database. 23,309 unigenes are common. Thus, 376 genes/proteins are additionally identified by TAIR10 database. Therefore, a total of 34,038 protein coding unigenes are mapped. This number is very close to the 35,938 genes reported in case of *P. patens*, another bryophyte with closest genome available^1^.

Mapping of unigenes at very low (10^−10^) e-value to different databases indicates the high quality of sequencing and assembly. Besides the mapped unigenes, we have found several sequences which do not show similarity with the database. Majority of such sequences are non-coding RNAs (ncRNAs)^10^. ncRNAs are reported to play significant role in transcriptional and post transcriptional gene regulation as well as in maintenance of genome stability^11^,^12^. However, there is also possibility of transcripts of uncharacterized genes in the sequence pool.

Annotated unigenes had been further used for functional annotation and classification using GO and KEGG, which provide information about probable biological functions and biosynthesis pathways^13^,^14^. Functional annotation of these unigenes has covered almost entire biological processes. GO analysis for biological processes shows that besides the protein metabolism, 6.6% unigenes are related to stress response and 6.4% to abiotic and biotic stimulus. This establishes the presence of stress tolerance mechanism in bryophytes.

Unigenes have also been mapped into 95 significant KEGG pathways; in which metabolism and biosynthesis of secondary metabolites pathways have higher representations. In secondary metabolites pathways, strong representation of genes for terpenoid, abscisic acid and flavonoids biosynthesis were observed (Supplementary Figure 1). These were supposed to be the major components of biotic and abiotic stress tolerance as well as thought to involve in signalling^14^.

Phenylpropanoids contribute to all aspects of plant responses towards biotic and abiotic stimuli. Phenylpropanoids are a diverse group of compounds derived from the carbon skeleton of phenylalanine that are involved in plant defense, structural support, and survival^15^. The pathway also gives rise to an array of other small molecules such as the flavonoids, coumarins, hydroxycinnamic acid conjugates, and lignans^15^,^16^. Flavonoids exhibit central functions in various aspects of plant life, and an enhanced amount of these compounds can be observed under different stress conditions. Highest log2 TPM valued Cinnamyl-alcohol dehydrogenase [EC: 1.1.1.195] converts aldehydes to alcohols which are the key substances for the production of final product in phenylpropanoid pathway. PRDX6; peroxiredoxin 6, 1-Cys peroxiredoxin [EC: 1.11.1.7] with log2 TPM value produces lignins in plants which is most important structural molecule although lignin is not present in bryophytes. Bryophytes do not synthesize lignin, but are widely found to accumulate soluble phenylpropanoids, such as flavonoids and lignans^17^,^18^. Phenylalanine ammonia-lyase [EC: 4.3.1.24] being the most enzyme of the phenylpropanoid pathway shows high TPM value. In qPCR expression analysis transcript of all the genes were observed and found to be following the similar pattern as observed in sequencing data.

We have analysed the representation of transcription factors (TFs) in *D. hirsuta* and found 447 TFs, representing 41 families. C2H2 and C3H TFs are highly represented followed by GRAS, bZIP and Homeobox. C2H2 and C3H are Zinc finger proteins and are known to be involved in various developmental pathways in plants^19^,^20^. Further, role of C2H2 in different biotic and abiotic stresses has also been reported^21^. GRAS family of proteins play major role in gibberellin (GA) signalling, which regulates plant growth and development^22^. They are also involved in Mycorrhizal signaling in legumes^23^. The basic leucine zipper (bZIP) families of transcription factors have several important roles in processes like development, general physiology and stress responses in all eukaryotes^24^. Homeobox family of proteins are also involved in the plant development especially in meristematic tissues^25^.

Bryophytes are the connecting link between aquatic and terrestrial plants^26^, thus sequences similarity of the annotated unigenes of *D. hirsuta* has been compared with some selected organisms from different phyla. *C. reinhardtii* from algae, *P. patens* from bryophytes, *S. moellendorffii* from pterydophytes and *A. thaliana* from angiosperms are selected for the homology analysis. Significant gene similarity is observed with phyla across the plant kingdom supporting bryophytes being the connecting link between aquatic and terrestrial plants. Maximum homology with *P. patens* is not surprising as both belong to bryophytes and closest to each other among the analysed organisms. We have found that about 30% of the annotated unigenes show similarity with algae *C. reinhardtii*, in which 133 matched uniquely. This indicates that the bryophytes retained several features from algae. About 60% genes are similar to *P. patens*, *S. moellendorffii* and *A. thaliana*, and about 10% annotated unigenes are exclusively shared with *P. patens.* These results indicate that the bryophytes have acquired several new features to adapt in new ecosystem. Most of these features are retained in higher plants in course of evolution. About 10% of the total unigens were exclusive to bryophytes, which might be related to their characteristic features. Since all of them are predicted or hypothetical proteins, we could not correlate them with functions. Characterization of bryophyte specific genes in depth will be taken up in future studies.

We have also analysed *D. hirsuta* transcriptome data for SSR markers. Being highly polymorphic, SSR markers are widely used in several evolutionary and genomics based studies^27^,^28^. High quality transcriptome data opens opportunity for marker discovery. We have identified 1594 SSRs, mostly dinucleotides and trinucleotide. Dinucleotides repeats are almost double of the trinucleotides. The result is in agreement with the earlier reports in higher plants^29^,^30^,^31^,^32^. AG/CT repeats are most abundant in dinucleotide SSRs, as reported in higher plants^33^,^34^. In trinucleotide repeats, AAG/CTT is most frequent and similar to the dicotyledonous plants^34^.

## Conclusion

Bryophytes occupy a very critical place in the evolution of terrestrial plants. *D. hirsuta* was selected as a representative species and a large transcriptome dataset was generated using 454 NGS technology. The data was used for functional annotation, gene ontology, elucidation of metabolic pathways, identification of transcription factor families and EST-SSRs markers. The study provides a comprehensive transcriptome data of rarely studied plant and would be useful for scientific community.

## Methods

### Plant materials

*D. hirsuta* was collected from Pachmarhi Biosphere Reserve Madhya Pradesh, India and conserved in moss house of National Botanical Research Insititute, Lucknow during this study.

### Library preparation and sequencing

Total RNA was prepared from the whole plant using Sigma Spectrum Plant Total RNA Isolation Kit and was further used for double stranded cDNA synthesis following the standard protocol. DS cDNA (5 μg) was purified using PCR purification column (Qiagen, GmbH, Germany). DNA was randomly sheared to an average fragment size 300–800 bp by applying 30 psi (2.1 bar) pressure of gaseous nitrogen for 1 minute using a nebulizer. The size of DNA fragments was analyzed on Agilent BioAnalyzer DNA 7500 Chip. Sheared cDNA ends were repaired by T4 DNA polymerase and T4 polynucleotide kinase. Small cDNA fragments were removed by Ampure beads purification. Sequencing library was prepared as per standard protocol (Roche, Switzerland) and the quality and quantity of the library was analysed on Agilent BioAnalyzer High sensitivity chip. An emulsion PCR based amplification of sequencing library was performed using GS emPCR kit and sequencing of the amplified fragments was done as per manufacture’s protocol on 454 Genome Sequencer FLX (Titanium) using half of the Pico Titer Plate (PTP, 70 × 75).

### *De novo* assembly

Reads with low quality score and less than 50 bp in length were removed before data processing (http://hiv.sanbi.ac.za/tools/qtrimwebcite). All the quality filtered reads were assembled using gsAssembler (Newbler v2.5.3) with the criteria of 50 bp overlap and 96% similarity. After assembly, both the contigs and singletons were pooled together to form a non-redundant unigenes set for further analysis.

### Annotations with different databases

All the unigenes (contigs and singletons) were searched for similarity against NCBI non-redundant protein database (nr; ftp://ftp.ncbi.nih.gov/blast/db/FASTA/) using Blastx with stringent parameters (e-value ≥ 10^−10^) and best hits were selected for analysis. These unigenes were also searched against the TAIR10 protein database (ftp://ftp.arabidopsis.org /home /tair/ Sequences/blastdatasets/ TAIR10_blastsets/) using Blastx with the same criteria. All the unigenes having similarity with TAIR10 protein database were assigned with the corresponding TAIR locus IDs.

### GO annotation and KEGG pathway analysis

GO analysis was performed with TAIR IDs assigned to unigenes for their functional categorization into different GO categories (cellular components, biological processes and molecular functions) using Gene Ontology annotations tool (http://www.arabidopsis.org/tools/bulk/go/index.jsp) available at TAIR.

For the pathway analysis, the TAIR IDs assigned to unigenes were used as inputs to obtain KO (KEGG Orthology) IDs from KAAS (KEGG Automatic Annotation Server; http://www.genome.jp/kegg/kaas/). The assigned KO IDs were used to identify pathways from KEGG server (Kyoto Encyclopedia of Genes and Genomes; http://www.genome.jp/kegg/tool/map_pathway1.html).

### Identification of transcription factors

Transcription factors encoding genes were searched using the similarity of unique sequences with *Arabidosis thaliana* transcription factor database available at http://arabidopsis.med.ohio-state.edu/AtTFDB/. All the identified transcription factors encoding genes were further classified into their corresponding families.

### Expression analysis of phenypropanoid pathway

To validate the expression of some selected genes of phenypropanoid pathway, quantitative real time PCR was performed in triplicates on ABI 7500 Fast Real-Time PCR machine using the SYBR Green PCR Master Mix (Applied Biosystems, CA, USA) following the standard protocol. Total RNA was isolated from gametophyte of *D. hirsuta* (100 mg) using Spectrum^TM^ plant total RNA kit (Sigma, USA). cDNA was synthesized from 2 μg of total RNA using first strand cDNA synthesis kit (Invitrogen, USA). Quality of cDNA was analysed by PCR amplification of actin gene. This cDNA was used for expression analysis of phenyl propanoid pathway genes. Primers used for real time PCR is provided in (Table S2). Amplification of actin was used as control.

### Similarity with other plant genomes

All the unigenes were compared for similarity with proteins sequences of *Chlamydomonas reinhardtii* (Algae, ftp://ftp.jgi-psf.org/pub/compgen/phytozome/v9.0/ Creinhardtii/annotation/), *Physcomitrella patens* (Bryophte, ftp://ftp.jgi-psf.org/pub/compgen/phytozome/v9.0/P*patens*_v1.6/annotation/), *Selaginella moellendorffii* (Pteridophyte, ftp://ftp.jgipsf.org/pub/compgen/phytozome/v9.0/Smoellendorffii/annotation/) and *A. thaliana* (Angiosperm) using Blastx with cut-off e-value of 1e-^10^ and venn diagram was made. Only best hits were considered for further analysis.

### Identification of EST-SSRs

All the unique sequences with more than 100 bp length were used for the SSRs identification. Identification of SSRs was performed using the microsatellite identification tool (MISA, http://pgrc.ipk-gatersleben.de/misa/misa.html). The criteria for the SSR search was repeat stretches having a minimum of five repeat units for dinucleotide (DNRs), trinucleotide (TNRs), tetranucleotide (TtNRs), pentanucleotide (PNRs) and hexanucleotide (HNRs). MNRs were excluded from the analysis. The maximum distance between two markers in a compound microsatellite was set to 100.

## Additional Information

**How to cite this article**: Singh, H. *et al.* Transcriptome sequencing of a thalloid bryophyte; *Dumortiera hirsuta* (Sw) Nees: assembly, annotation, and marker discovery. *Sci. Rep.* **5**, 15350; doi: 10.1038/srep15350 (2015).

## Supplementary Material

Supplementary Information

Supplementary Information

Supplementary Information

Supplementary Information

Supplementary Information

## Figures and Tables

**Table 1:** Details of *D. hirsuta* transcriptome sequencing using 454 GS FLX pyrosequencer.

Total reads generated	548123
Total Bases generated (Mb)	195.3
Average read length (bases)	356.8
Longest read length (bases)	737
Short reads (<50 bases)	1968
Reads of ribosomal origin	121117
Total reads discarded	123085
Total quality passed (excluding ribosomal and <50 bases)	425038

**Table 2:** *De novo* assembly outputs of *D. hirsuta* transcriptome using gsAssembler (Newbler v2.5.3).

Reads used for assembly (excluding ribosomal and <50 bases)	425038
All Contigs (≥100 bp)	12439
Large contigs (≥500 bp)	8007
Singletons	72801
Average contig size (bp)	989
Largest contig Size (bp)	4843
N50 contig size^a^ (bp)	1071
% Reads Aligned	80.7
Q40 Plus bases^b^	95.44

^a^N50 corresponds to the length of the smallest contig in the set of largest contigs whose combined length represents 50% of the total assembly size.

^b^Q40Plus bases percentage of bases called that have a quality score of 40 or above.

**Table 3:** Details of annotation of *D. hirsuta* unigenes with different public databases and pathway analysis.

Number of unigenes (contigs and singletons)	85240
Total hits with NCBI NR database	33662
Unique hits with NCBI NR database	20760
Total hits with TAIR 10 protein database	23685
Unique hits with TAIR 10 protein database	9014
GO Annotation	
Cellular component	20582
Molecular Function	14493
Biological Process	27610
Locus with KO ids assigned	4305
KEGG pathways matched	95

**Table 4:** Major enzymes of phenylpropanoid pathway expressing in *D. hirsuta.*

Contigs	Locus ID	TPM	Annotation	KEGG ID
contig04402	AT4G37990.1	7.77	cinnamyl-alcohol dehydrogenase [EC:1.1.1.195]	K00083
contig02754	AT1G48130.1	7.27	peroxiredoxin 6, 1-Cys peroxiredoxin [EC 1.11.1.15]	K00430
contig01164	AT3G53260.1	6.27	phenylalanine ammonia-lyase [EC:4.3.1.24]	K00487
contig07293	AT1G51680.1	6.04	4-coumarate–CoA ligase [EC:6.2.1.12]	K00588
contig02858	AT5G48930.1	5.69	shikimate O-hydroxycinnamoyltransferase [EC:2.3.1.133]	K01188
contig07426	AT1G14540.1	4.93	peroxidase [EC:1.11.1.7]	K01904
contig04721	AT4G34050.1	4.81	caffeoyl-CoA O-methyltransferase [EC:2.1.1.104]	K05349
contig04608	AT1G15950.1	4.81	cinnamoyl-CoA reductase [EC:1.2.1.44]	K09753
contig04082	AT5G20950.2	4.55	beta-glucosidase [EC:3.2.1.21]	K09754
contig05783	AT2G40890.1	4.55	coumaroylquinate 3′-monooxygenase [EC 1.14.13.36]	K10775
contig05872	AT2G30490.1	4.04	trans-cinnamate 4-monooxygenase [EC:1.14.13.11]	K11188
