# Supplementary material for: Transcriptome sequencing of a thalloid bryophyte; Dumortiera hirsuta (Sw) Nees: assembly, annotation, and marker discovery
Source: Sci Rep. 2015 Oct 20;5:15350. doi: 10.1038/srep15350 (PMC4611483; doi:10.1038/srep15350)

Transcriptome sequencing of a thalloid bryophyte; *Dumortiera hirsuta* (Sw) Nees: assembly, annotation, and marker discovery

Harpal Singh[1], Krishan Mohan Rai[1], Santosh Kumar Upadhyay[2], Poonam Pant[1], Praveen Chandra Verma[1], Ajit Pratap Singh[1], Pradhyumna Kumar Singh[1]*

[1]CSIR-National Botanical Research Institute, Council of Scientific and Industrial Research, Rana Pratap Marg, Lucknow 226001, UP, India

[2]Department of Botany, Kashyap Block, Panjab University, Chandigarh, 160014-UT, India

*Corresponding Author
Dr. Pradhyumna Kumar Singh
Senior Scientist
CSIR-National Botanical Research Institute,
Council of Scientific and Industrial Research,
Rana Pratap Marg, Lucknow-226001, India
Tel: +91-522-2297942
Fax: +91-522-205836, 2205839
E-mail: pksingh@nbri.res.in; pradhyumnasingh@hotmail.com

Supplemental Data 3. GO functional annotation details of *Dumortiera hirsuta* unigenes.

Keyword Category	Functional Category	Gene Count	Gene Count %
GO Cellular Component	other cytoplasmic components	3414	16.5873093
	other intracellular components	2990	14.52725683
	nucleus	2685	13.04537946
	chloroplast	2166	10.52375862
	other membranes	1952	9.484015159
	plasma membrane	1403	6.816635895
	cytosol	1148	5.577689243
	mitochondria	1078	5.23758624
	plastid	999	4.853755709
	extracellular	644	3.128947624
	other cellular components	547	2.657662035
	Golgi apparatus	515	2.502186376
	ER	342	1.661646099
	ribosome	288	1.399280925
	cell wall	249	1.209794966
	unknown cellular components	162	0.78709552
GO Molecular Function	other binding	2570	17.73269854
	other enzyme activity	1759	12.13689367
	nucleotide binding	1651	11.39170634
	hydrolase activity	1403	9.680535431
	unknown molecular functions	1389	9.583937073
	transferase activity	1288	8.88704892
	protein binding	1111	7.665769682
	DNA or RNA binding	826	5.699303112
	transporter activity	644	4.44352446
	kinase activity	527	3.636238184
	other molecular functions	371	2.559856482
	nucleic acid binding	345	2.380459532
	structural molecule activity	317	2.187262817
	transcription factor activity	235	1.621472435
	receptor binding or activity	57	0.393293314
GO Biological Process	other cellular processes	5762	20.86925027
	other metabolic processes	5339	19.33719667
	protein metabolism	2262	8.19268381
	response to stress	1831	6.631655197
	cell organization and biogenesis	1775	6.428830134
	response to abiotic or biotic stimulus	1771	6.414342629
	transport	1657	6.00144875
	developmental processes	1653	5.986961246
	unknown biological processes	1600	5.795001811
	other biological processes	1534	5.555957986
	transcription,DNA-dependent	762	2.759869612
	signal transduction	740	2.680188338
	electron transport or energy pathways	482	1.745744296
	DNA or RNA metabolism	442	1.60086925

Supplemental Data 4. Details of KEGG pathways identified in *Dumortiera hirsuta* unigenes.

S. No.	KO ids	Pathways	No. Of Genes
1	ko01100	Metabolic pathways	721
2	ko01110	Biosynthesis of secondary metabolites	309
3	ko03040	Spliceosome	92
4	ko00190	Oxidative phosphorylation	87
5	ko03013	RNA transport	80
6	ko00230	Purine metabolism	72
7	ko04141	Protein processing in endoplasmic reticulum	70
8	ko00240	Pyrimidine metabolism	61
9	ko03008	Ribosome biogenesis in eukaryotes	52
10	ko04120	Ubiquitin mediated proteolysis	49
11	ko03018	RNA degradation	44
12	ko00195	Photosynthesis	40
13	ko03015	mRNA surveillance pathway	39
14	ko04110	Cell cycle	37
15	ko00520	Amino sugar and nucleotide sugar metabolism	35
16	ko03050	Proteasome	34
17	ko00330	Arginine and proline metabolism	33
18	ko04111	Cell cycle - yeast	32
19	ko04146	Peroxisome	31
20	ko03420	Nucleotide excision repair	30
21	ko00010	Glycolysis / Gluconeogenesis	29
22	ko00260	Glycine, serine and threonine metabolism	29
23	ko00500	Starch and sucrose metabolism	29
24	ko00510	N-Glycan biosynthesis	29
25	ko00564	Glycerophospholipid metabolism	29
26	ko00900	Terpenoid backbone biosynthesis	29
27	ko03022	Basal transcription factors	29
28	ko04142	Lysosome	29
29	ko00620	Pyruvate metabolism	28
30	ko04075	Plant hormone signal transduction	28
31	ko00270	Cysteine and methionine metabolism	27
32	ko01210	2-Oxocarboxylic acid metabolism	27
33	ko00250	Alanine, aspartate and glutamate metabolism	26
34	ko03060	Protein export	26
35	ko00710	Carbon fixation in photosynthetic organisms	25
36	ko00860	Porphyrin and chlorophyll metabolism	25
37	ko00970	Aminoacyl-tRNA biosynthesis	25
38	ko04114	Oocyte meiosis	25
39	ko03020	RNA polymerase	24
40	ko00630	Glyoxylate and dicarboxylate metabolism	23
41	ko04113	Meiosis - yeast	23

42	ko00400	Phenylalanine, tyrosine and tryptophan biosynthesis	22
43	ko00561	Glycerolipid metabolism	22
44	ko00680	Methane metabolism	22
45	ko04721	Synaptic vesicle cycle	22
46	ko00513	Various types of N-glycan biosynthesis	21
47	ko04151	PI3K-Akt signaling pathway	20
48	ko04626	Plant-pathogen interaction	20
49	ko00020	Citrate cycle (TCA cycle)	19
50	ko00280	Valine, leucine and isoleucine degradation	19
51	ko00563	Glycosylphosphatidylinositol (GPI)-anchor biosynthesis	19
52	ko00562	Inositol phosphate metabolism	18
53	ko03030	DNA replication	18
54	ko03410	Base excision repair	18
55	ko00051	Fructose and mannose metabolism	17
56	ko00350	Tyrosine metabolism	17
57	ko03440	Homologous recombination	17
58	ko00130	Ubiquinone and other terpenoid-quinone biosynthesis	16
59	ko00480	Glutathione metabolism	16
60	ko00640	Propanoate metabolism	16
61	ko00906	Carotenoid biosynthesis	16
62	ko03430	Mismatch repair	16
63	ko04070	Phosphatidylinositol signaling system	16
64	ko04130	SNARE interactions in vesicular transport	16
65	ko04310	Wnt signaling pathway	16
66	ko04810	Regulation of actin cytoskeleton	16
67	ko00030	Pentose phosphate pathway	15
68	ko00100	Steroid biosynthesis	15
69	ko00360	Phenylalanine metabolism	15
70	ko00910	Nitrogen metabolism	15
71	ko04066	HIF-1 signaling pathway	15
72	ko04712	Circadian rhythm - plant	15
73	ko04914	Progesterone-mediated oocyte maturation	15
74	ko00052	Galactose metabolism	14
75	ko00053	Ascorbate and aldarate metabolism	14
76	ko00410	beta-Alanine metabolism	14
77	ko00770	Pantothenate and CoA biosynthesis	14
78	ko00940	Phenylpropanoid biosynthesis	14
79	ko01040	Biosynthesis of unsaturated fatty acids	13
80	ko04623	Cytosolic DNA-sensing pathway	13
81	ko05034	Alcoholism	13
82	ko00061	Fatty acid biosynthesis	12
83	ko00196	Photosynthesis - antenna proteins	12
84	ko00600	Sphingolipid metabolism	12
85	ko00720	Carbon fixation pathways in prokaryotes	12
86	ko00040	Pentose and glucuronate interconversions	11
87	ko00592	alpha-Linolenic acid metabolism	11
88	ko00760	Nicotinate and nicotinamide metabolism	11
89	ko00071	Fatty acid metabolism	10

90	ko00290	Valine, leucine and isoleucine biosynthesis	10
91	ko00340	Histidine metabolism	10
92	ko00650	Butanoate metabolism	10
93	ko00920	Sulfur metabolism	10
94	ko00941	Flavonoid biosynthesis	10
95	ko04530	Tight junction	10

Supplemental Table 1. Details of SSRs mining from *Dumortiera hirsuta* unigenes.

**Details of SSRs mining**	**Numbers**
Total number of sequences examined	81838
Total size of examined sequences (bp)	32898624
Total number of identified SSRs	1594
Number of SSR containing sequences	1479
Number of sequences containing more than 1 SSR	102
Number of SSRs present in compound formation	89
SSRs motif identified	
DNRs	1040
TNRs	490
TtNRs	43
PNRs	14
HNRS	7

Table S2: Primers used for Real Time Q-PCR

Gene	Forward (5'-3')	Reverse (5'-3')
Cinnamyl-alcohol dehydrogenase	AGCAACTGCAGGGTTGACAGT	TCACAGGAGCAGGGACCATAC
Peroxiredoxin 6, 1-Cys peroxiredoxin	CCCAGGGAGGTGGACTATTTTC	GCAGAGACTAGCTCTACGCGAGTT
Phenylalanine ammonia-lyase	TCTCCTGTGGTTTCAGCATTCA	ATGTCAGGAGCCAGTGTGTTGT
4-coumarate-coA ligase	TTTGTTCACTCTCTGGGATTTTACG	GTTCCATCAGCCAGCGTGTT
Shikimate O-hydroxycinnamoyl transferase	TAAGCCCGGAGATTTCACAAGT	AGCAGCCGCGGTAATACGTA
Peroxidase	CTCCTCGATCCCTGCTTTAGC	GGTGATGGCATACCGGTTTG
Caffeoyl-coA O-methyltransferase	CATCACGACTTCCAACCACACT	CTTAGGCTCGGGCTTCTTCTTT
Cinnamoyl-coA reductase	AATCTATGAAAGGGCTACGCAAA	GCCGACTTCCCTTACCTACATTG
Beta-glucosidase	GCCGTGGATGTATCACATGCTA	GCTGGTATGGCTTCAAAATCTGT
Coumaroylquinate 3'-monooxygenase	CTCACCGACAGCCACCTTATC	GTGGGCAGATGACGAGAGAATT
Trans-cinnamate 4-monooxygenase	AGCATCAGAGGGCTGTTGGA	TTCACTCTGAGGATACGCTGTTG
Actin	GCTGCACGCCACCTTATTG	GGCAAGTACCTGCATCATTCAC

Figure S1 Phenylpropanoid pathway; Highligted in Red showing enzymes found in *D. hirsuta*

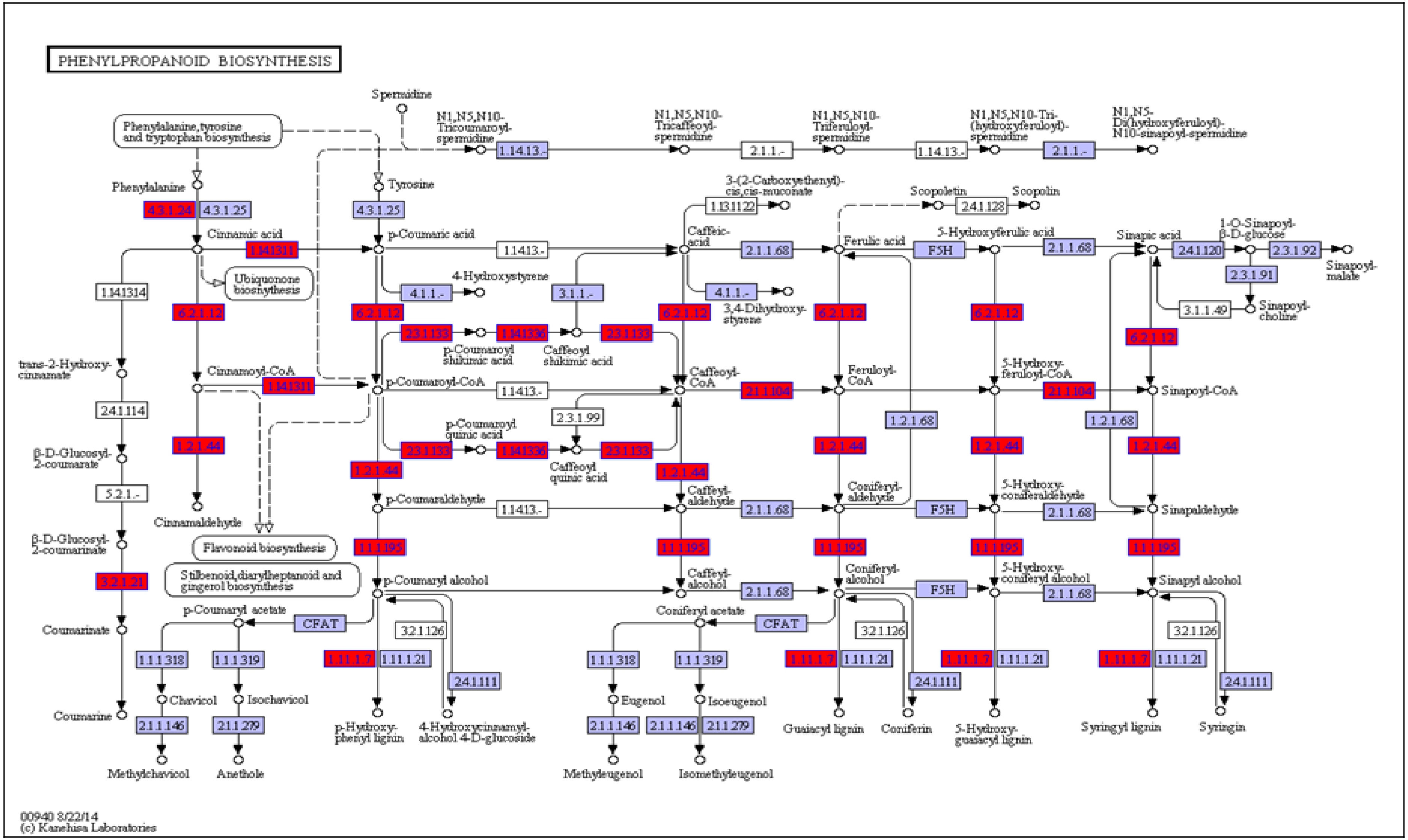

Supplement: Supplementary Information [file srep15350-s1.pdf]
